# Morbilliform Rashes in a Patient with COVID-19 Infection: A Case Report

**DOI:** 10.31729/jnma.5128

**Published:** 2021-04-30

**Authors:** Kiran Ghimire, Nabin Adhikari

**Affiliations:** 1 Shree Birendra Hospital, Chhauni, Kathmandu, Nepal

**Keywords:** *coagulopathy*, *COVID-19*, *cytokine release syndrome*, *D-dimer*, *morbilliform*

## Abstract

As the current COVID-19 pandemic is evolving, skin lesions are being reported more, the most common skin manifestation being morbilliform rashes. We describe a patient of severe COVID-19 infection, 48-year-old who initially presented with fever, cough and constitutional symptoms who developed morbilliform macular rashes during his illness. The rash appeared on 6th day of illness in the trunk, arms with sparing of palms and soles, associated with itching. He later developed features of the cytokine-storm syndrome. The exact mechanism for the rashes is yet to be elaborated, however, it is postulated that it is either due to immune-mediated vasodilation or micro thrombosis secondary to low-grade-coagulopathy associated with COVID-19. Recognition of rashes as a feature of this disease is particularly significant to clinicians as it aids in early diagnosis, particularly in resource-poor countries. There is no evident association, however, between the severity and the rashes in COVID-19 infection.

## INTRODUCTION

An epidemic of unexplained respiratory infections was reported in Wuhan, China in December 2019; similar to viral pneumonia.^1^ A new coronavirus was identified after analyses of respiratory samples of patients, and named SARS-CoV-2; the disease was later named Coronavirus disease 2019 (COVID-19).^1^ As the pandemic evolved, various types of skin lesions were described.2 Skin lesions are fairly common than was generally understood. Recalcati et al, reported 20.4% of COVID-19 patients developed skin manifestations.^3^ The most common skin lesion was morbilliform maculopapular rashes, present in 36.1% of patients.^4^ We describe a patient of COVID-19 infection with generalized morbilliform rashes who subsequently developed the cytokine-storm syndrome.

## CASE REPORT

A 48-year-old warrant officer with no chronic illness, presented to us with initial complains of fever, cough, and fatigue for 2 days. At admission, his temperature was 100.3^o^F, pulse rate was 90 beats per minute, oxygen saturation by pulse oximeter was 97% at room air with a respiratory rate of 22 breaths per minute. His blood pressure was 100/70mmHg. He had a persistent fever on subsequent days. We added amoxiclav to his symptomatic treatment. He became afebrile for the next 3 days but had a persistent cough and asthenic symptoms.

He again developed a fever on 5th day. He had dyspnoea on exertion with oxygen saturation 92% without supplementation. On examination, he was ill-looking and dyspnoeic, lung auscultation revealed bilateral crackles; the remaining examination was unremarkable. COVID-19 infection was suspected and treatment started with intramuscular artemether (as he did not tolerate chloroquine), azithromycin, vitamin C and zinc. His blood tests showed lymphopenia (lymphocytes: 1392/mm^3^), high erythrocyte sedimentation rate (35mm in 1st hour) with high transaminases (2-3 x upper limit of normal range). Chest X-ray showed mediastinal lymphadenopathy with interstitial infiltrates. His pharyngeal swab results came “positive” for COVID-19. He had few episodes of loose stools during the initial days of illness, which later subsided.

On 6^th^ day of illness, he developed papular-to-plaque rashes over his arms and legs, which progressed to the abdomen and later generalized all over his body.

**Figure 1.**
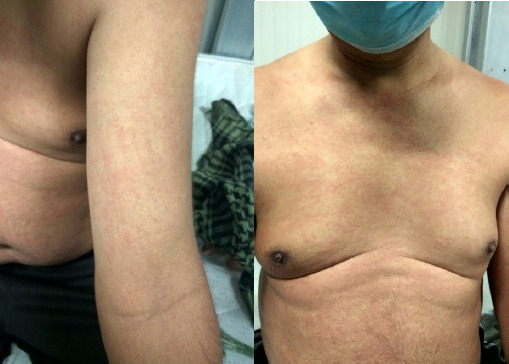
Erythematous, blanching rashes in abdomen and in arms in a patient with severe COVID infection.

The rashes were erythematous, diffuse, blanching, morbilliform (Figure 1).

Eventually, the rashes were distributed on the neck, thorax, abdomen, buttocks, extremities, including folds and scalp, sparing the palmoplantar region and mucosa. Over a couple of days, the lesions became itchier, while the erythema intensity decreased. After three days, the rashes began to fade and completely disappeared on the fourth day of the appearance of the rash.

However, he had a persistent fever even after 10 days of illness. Then, injection ciprofloxacin was started on 11^th^ day along with thromboprophylaxis after which his medical evacuation was done to a tertiary hospital in Bamako where his detailed evaluation was done, which showed leucocytosis (WBC-14800/mm^3^), high CRP (288mg/L), ferritinemia (>2000ng/ml). He also had high D-dimer (D-dimer-2.34ug/ml; normal <0.5). These features suggested a severe COVID-19 infection with the features of the cytokine storm. Injection methylprednisolone 120mg per day was started. Antibiotics upgraded to imipenem/meropenem 1gm every 8 hourly, in addition to hydroxychloroquine plus azithromycin. He had normal chest and abdominal scans. His clinical picture improved significantly. His pharyngeal swab for COVID-19 was negative on the subsequent two samples. Repeat chest and abdominal scans were unremarkable. He had high levels of IgG for COVID-19 at the time of discharge.

## DISCUSSION

We describe a case of a 48-year-old with COVID infection with fever and cough who subsequently developed features of the cytokine storm. He had a prolonged duration of fever-documented for 10 days present throughout the day. He developed rashes all over his body on 6^th^ day of illness. These rashes were associated with high-grade fever, however no mucosal lesions, and showed palmoplantar sparing.

During the initial days of this pandemic, skin rashes were rarely described, however as the pandemic evolved, these are being increasingly reported. The literature review done by the authors noted that skin manifestations of COVID-19 are common. Recalcati reports in a series of 88 COVID-19 patients, 18 developed skin manifestations (20.4%) erythematous rash, generalized urticaria and varicelliform rash. All of them pruritic in mild intensity, resolving within a few days.^3^ Among 15 patients with COVID-19 infection, the authors noted two patients of morbilliform skin rashes, both aged >40 years consistent with a study by Sachdeva, et al.^4^ where the mean patient age was 53.6 years. The authors noted morbilliform rashes in a severe COVID-19 patient, however, the correlation of rashes with disease severity cannot be unquestionably delineated.

The exact mechanism of rashes in COVID-19 infection is yet to come out, however, it is postulated that the viral infection activates Langerhans cell resulting in vasodilation.^5^ Also, low-grade coagulopathy associated with this infection leading to micro thrombosis in small vessels may explain such generalized morbilliform rashes,^6^ as evident in this patient too. It is more likely that a combination of such mechanisms is responsible for the rashes in COVID-19 positive patients.

In the event of this pandemic, especially in developing countries, where resources are particularly low, identification of skin manifestations can aid in the diagnosis of COVID-19 infection, along with other clinical features. As COVID-19 has a tendency to produce asymptomatic cases for up to 14 days after infection, skin manifestations may serve as an indicator of infection, aiding in timely diagnosis.^4^

One of the limitations of our study was follow up of the patient as he was evacuated to a tertiary hospital, and subsequent clinical assessment was indirectly conducted.

The occurrence of skin manifestations is common in COVID-19 infections, consistent with the current literature. However, no association is evident with the severity of illness and the rashes in general. These inferences require further systematic studies to demonstrate and explicate an understanding of COVID-19 related skin manifestations, and especially its association with severity of infection.
